# Supplementary figures and images for: Characterizing Spatially Continuous Variations in Tissue Microenvironment through Niche Trajectory Analysis
Source: bioRxiv. 2024 Apr 28:2024.04.23.590827. Preprint. [Version 1] doi: 10.1101/2024.04.23.590827 (PMC11071437; doi:10.1101/2024.04.23.590827)

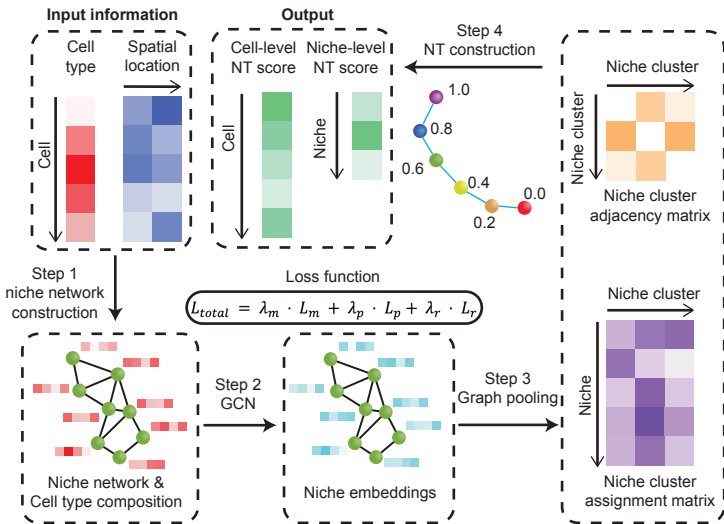

Supplement: Supplement 4 [file media-4.pdf]

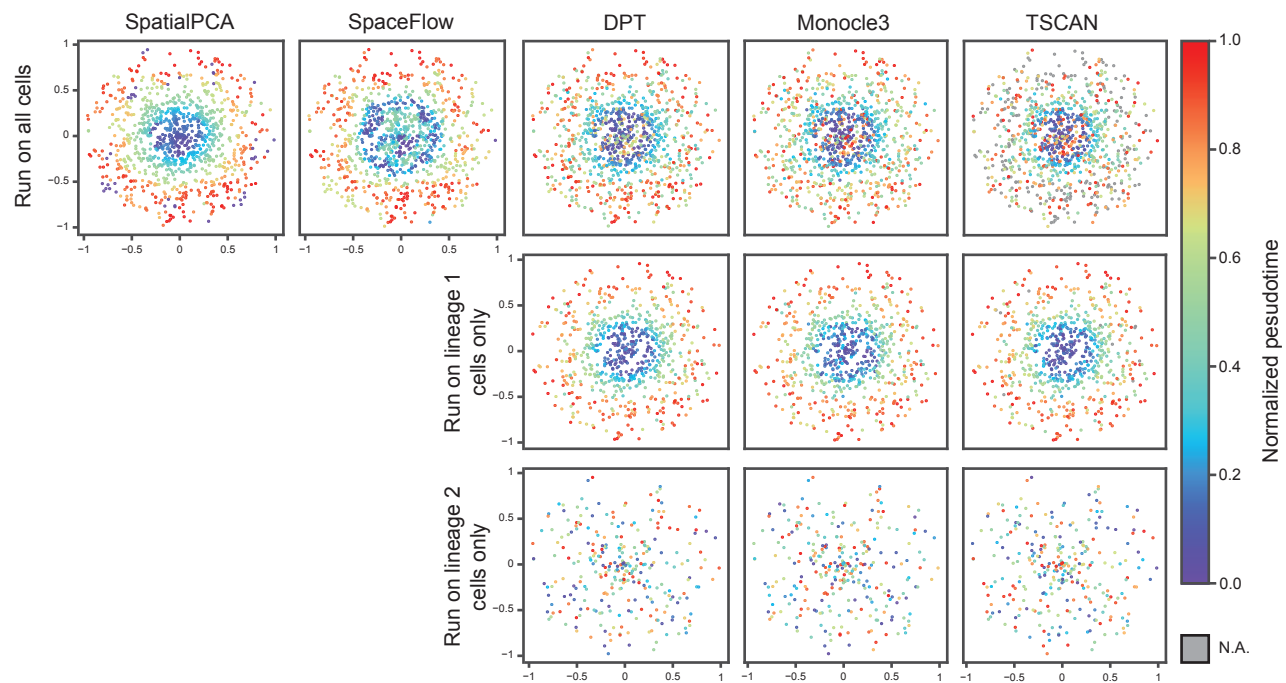

Supplement: Supplement 5 [file media-5.pdf]

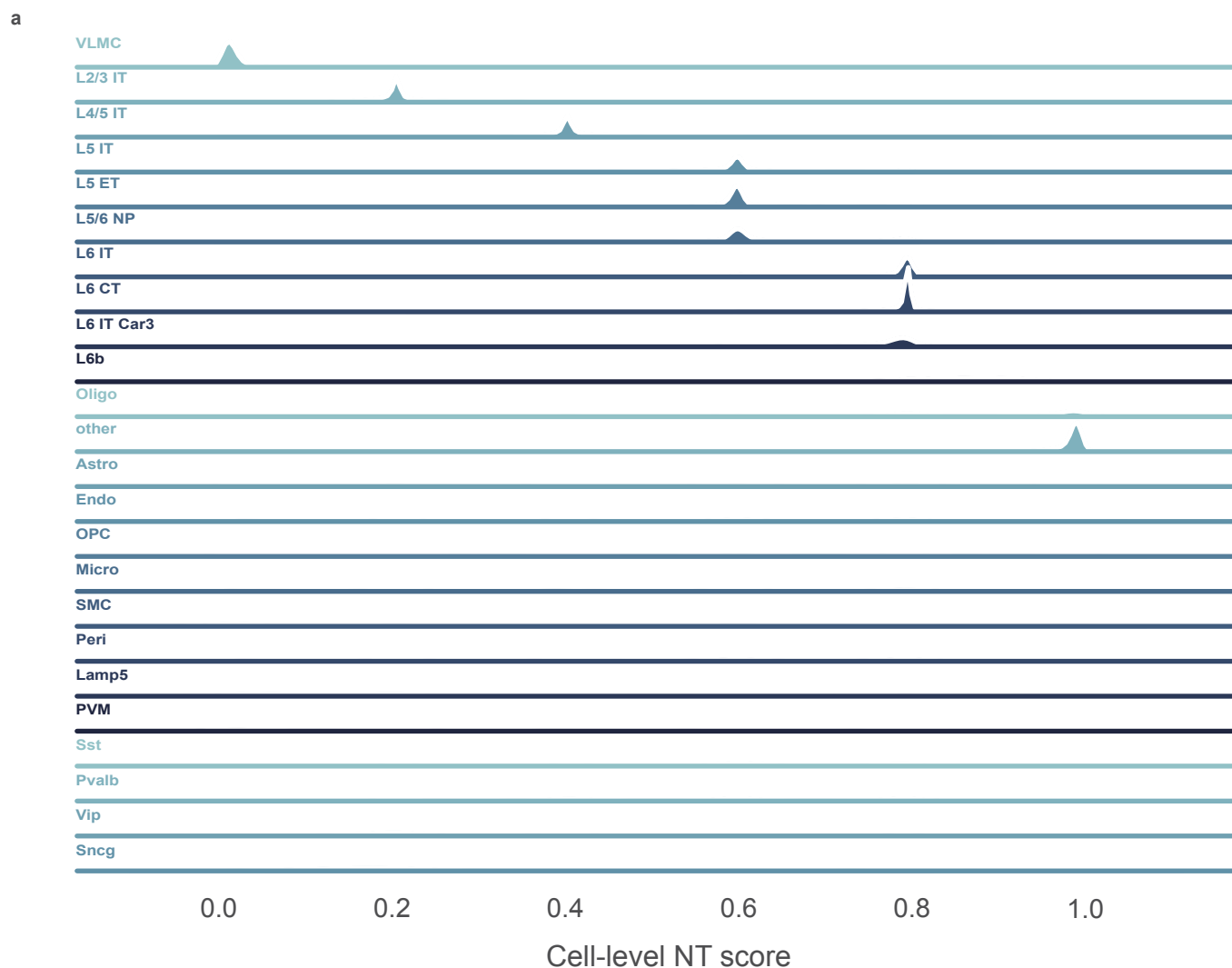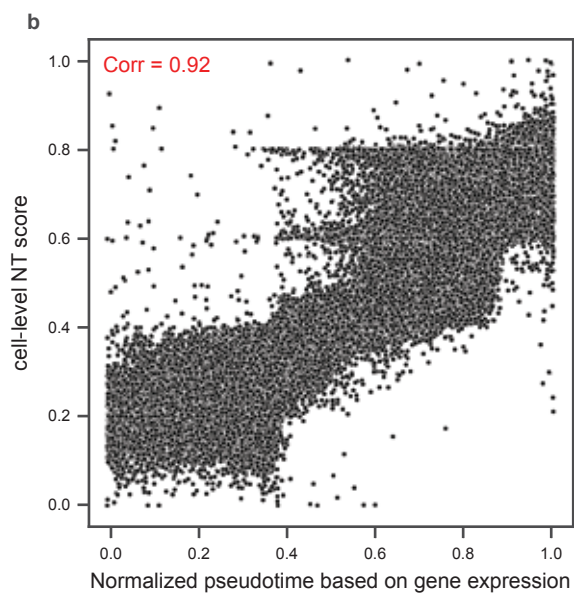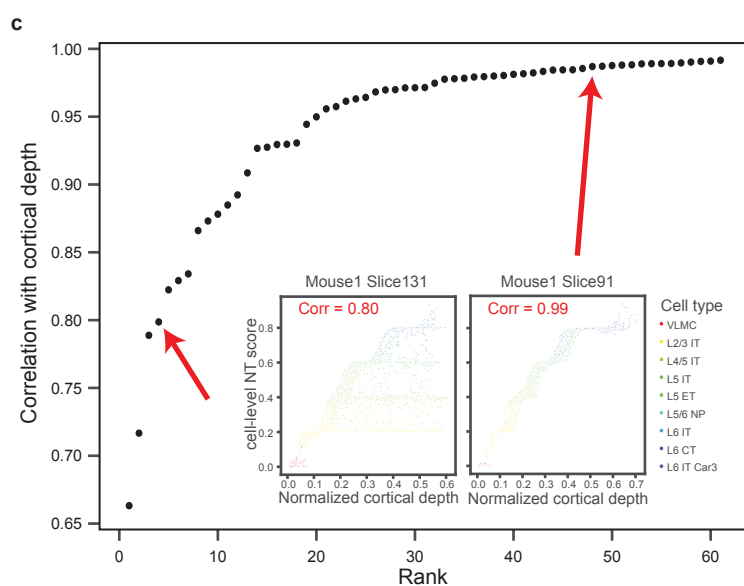

Supplement: Supplement 6 [file media-6.pdf]

**a**

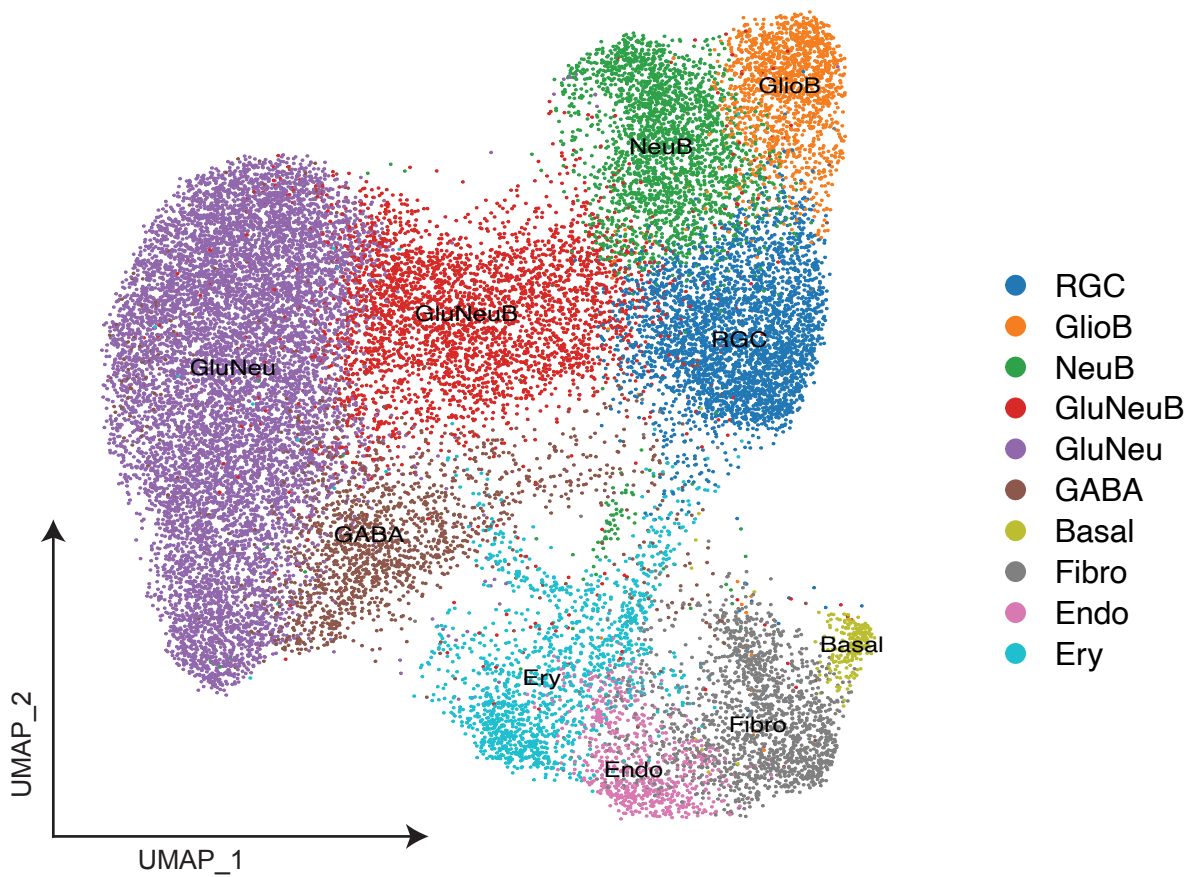**b**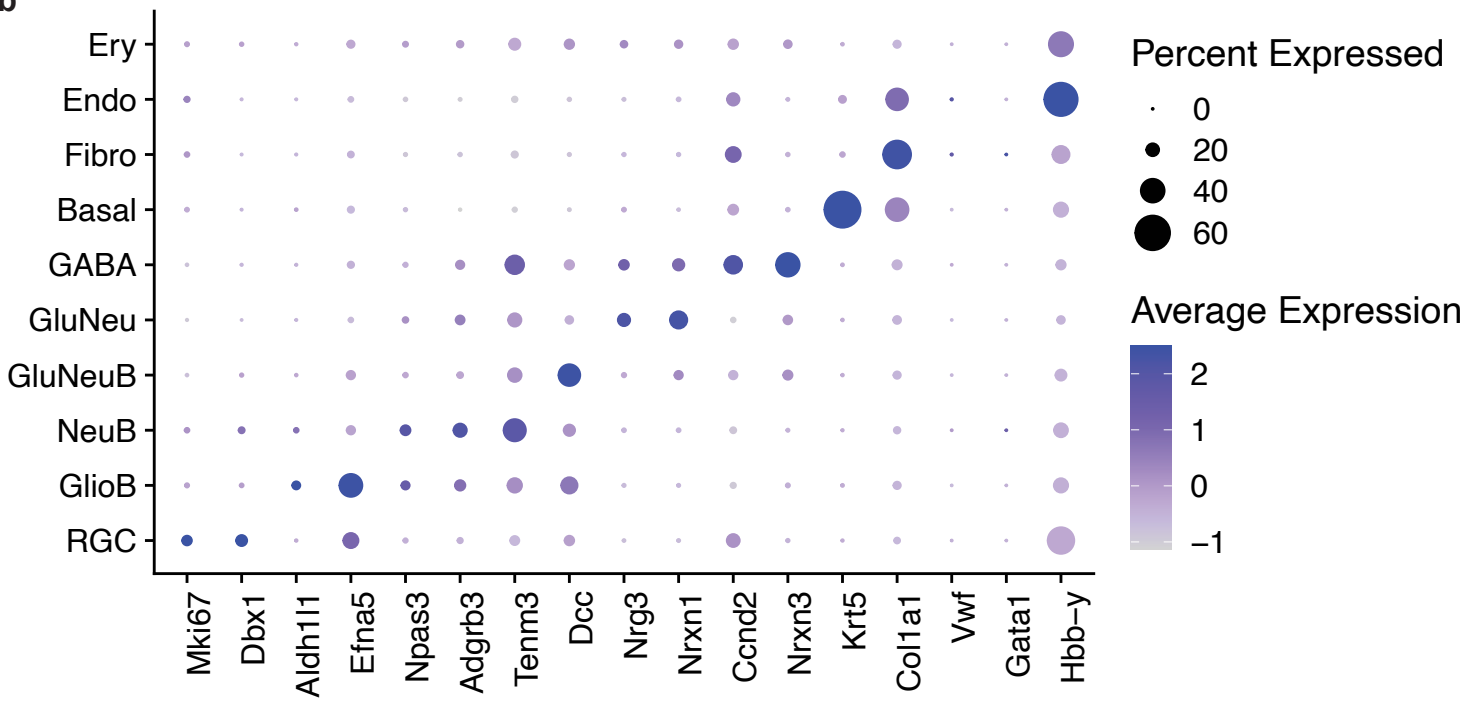

Supplement: Supplement 7 [file media-7.pdf]

E12.5

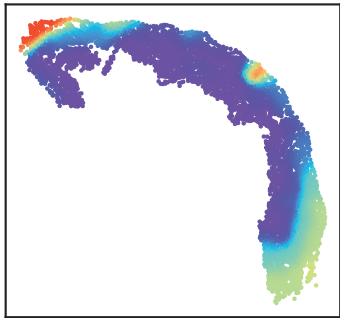

E16.5

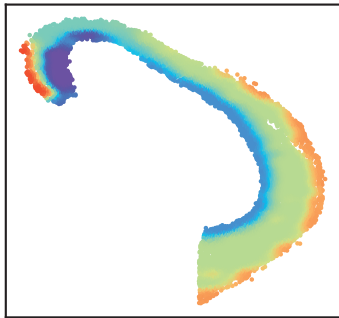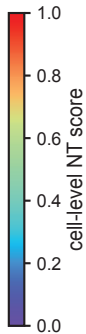

Supplement: Supplement 8 [file media-8.pdf]

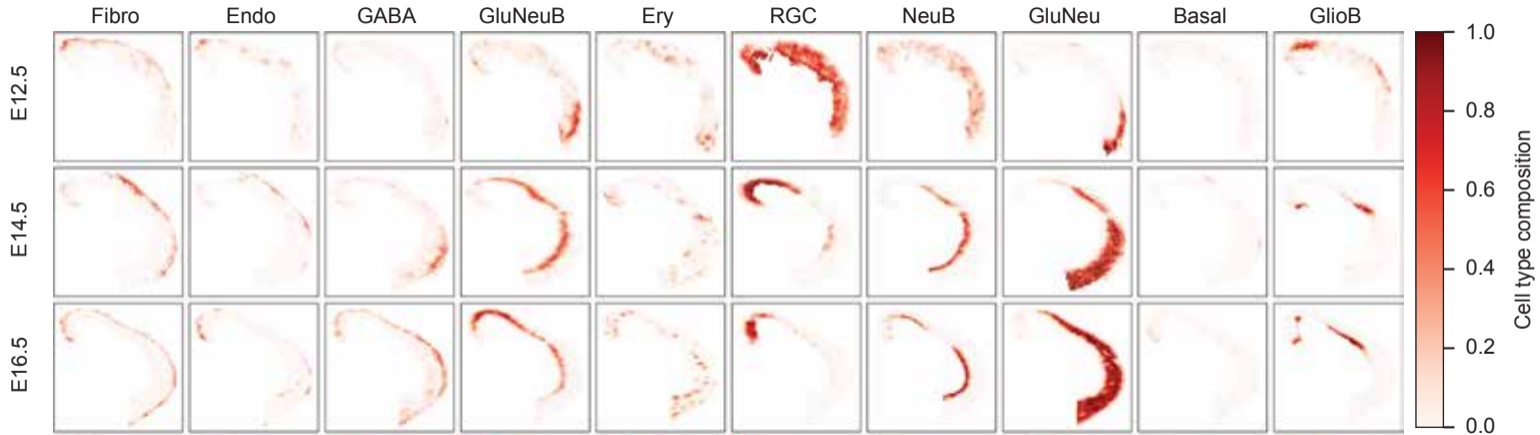

Supplement: Supplement 9 [file media-9.pdf]

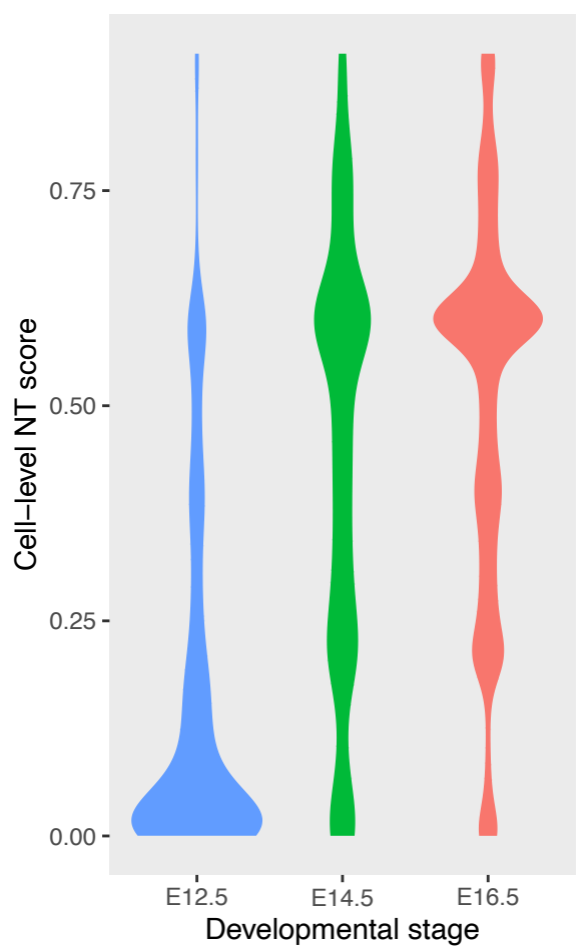

Supplement: Supplement 10 [file media-10.pdf]

a

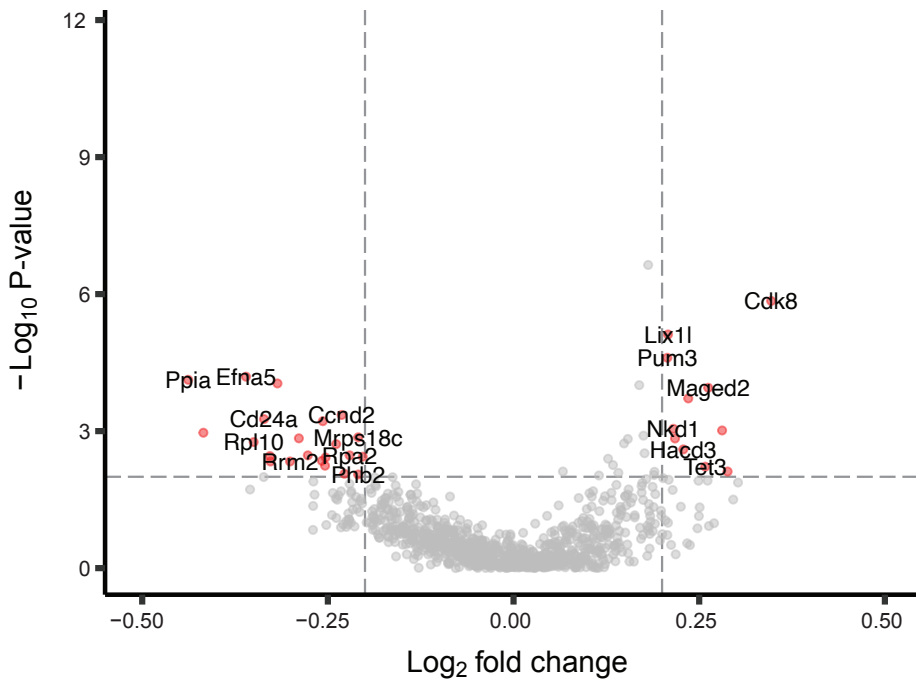

b

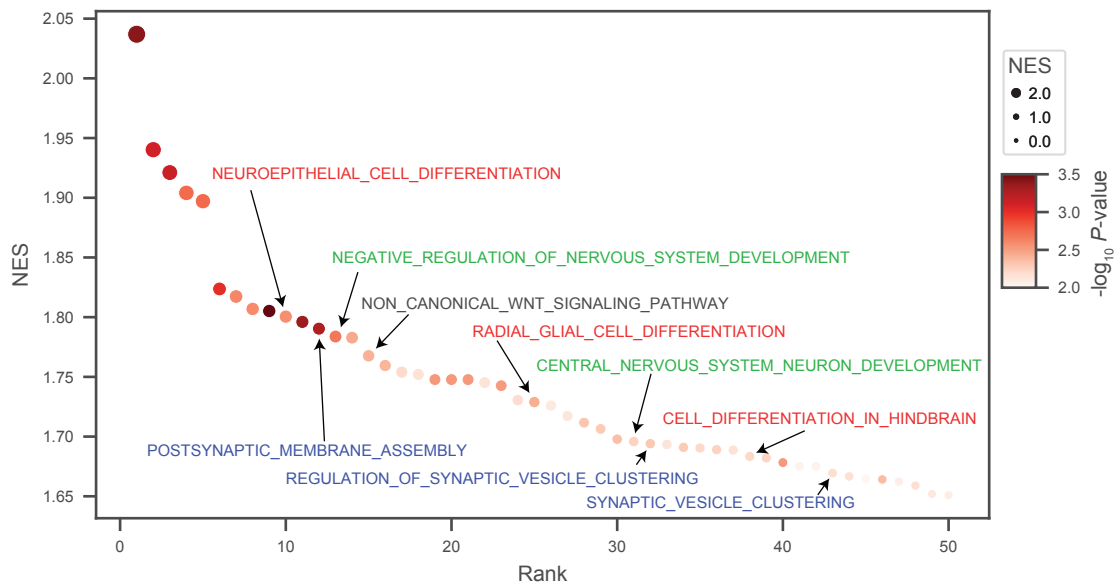

Supplement: Supplement 11 [file media-11.pdf]

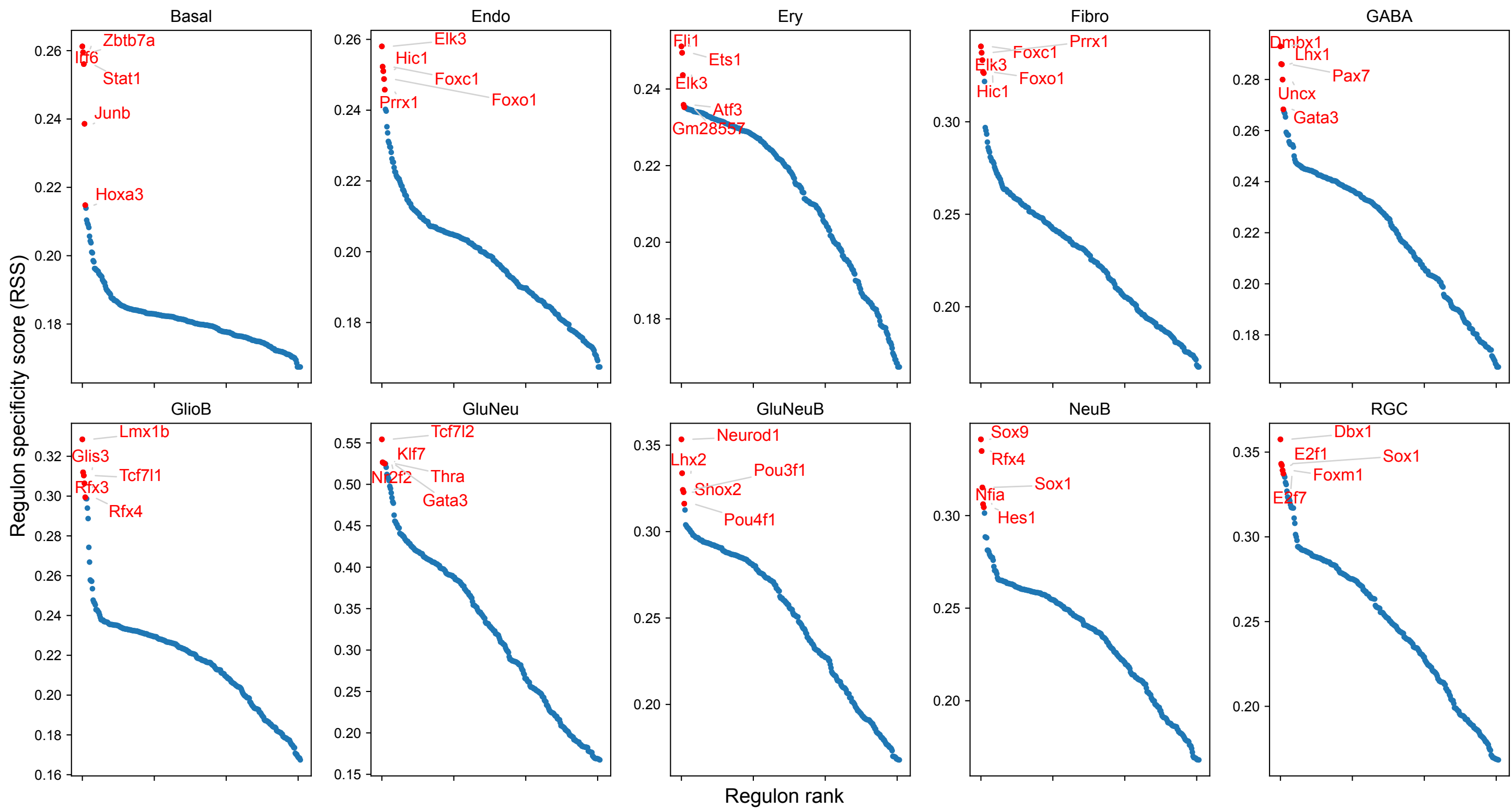

Supplement: Supplement 12 [file media-12.pdf]

NT-Low

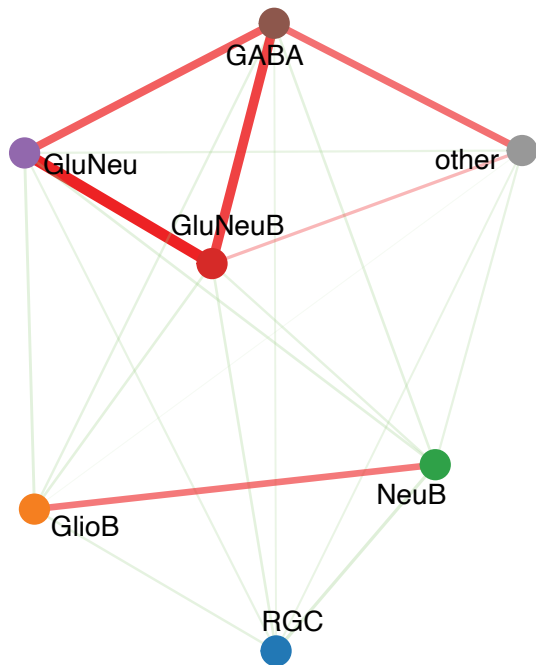

NT-High

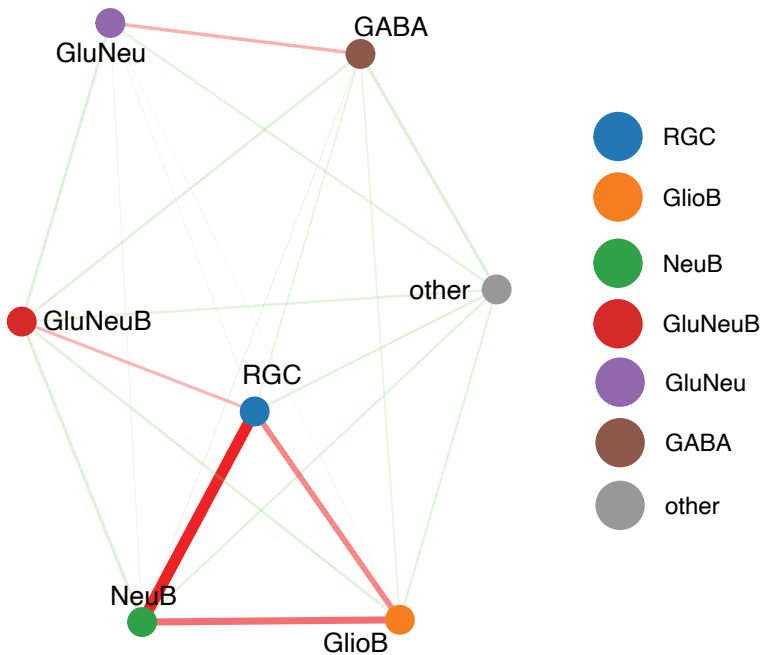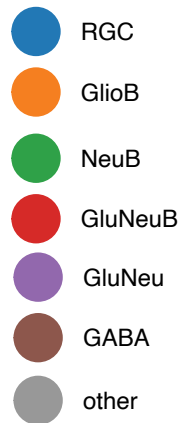

Supplement: Supplement 13 [file media-13.pdf]
